# Behavioral and genetic evidence for a novel animal model of Attention-Deficit/Hyperactivity Disorder Predominantly Inattentive Subtype

**DOI:** 10.1186/1744-9081-4-56

**Published:** 2008-12-01

**Authors:** T Sagvolden, T DasBanerjee, Y Zhang-James, FA Middleton, SV Faraone

**Affiliations:** 1 Department of Physiology, Institute of Basic Medical Sciences, University of Oslo, Oslo, Norway; 2 Department of Neuroscience & Physiology, Upstate Medical University, Syracuse, NY, USA; 3 Department of Psychiatry, Upstate Medical University, Syracuse, NY, USA

## Abstract

**Background:**

According to DSM-IV there are three subtypes of Attention-Deficit/Hyperactivity Disorder, namely: ADHD predominantly inattentive type (ADHD-PI), ADHD predominantly Hyperactive-Impulsive Type (ADHD-HI), and ADHD combined type (ADHD-C). These subtypes may represent distinct neurobehavioral disorders of childhood onset with separate etiologies. The diagnosis of ADHD is behaviorally based; therefore, investigations into its possible etiologies should be based in behavior. Animal models of ADHD demonstrate construct validity when they accurately reproduce elements of the etiology, biochemistry, symptoms, and treatment of the disorder. Spontaneously hypertensive rats (SHR) fulfill many of the validation criteria and compare well with clinical cases of ADHD-C. The present study describes a novel rat model of the predominantly inattentive subtype (ADHD-PI).

**Methods:**

ADHD-like behavior was tested with a visual discrimination task measuring overactivity, impulsiveness and inattentiveness. Several strains with varied genetic background were needed to determine what constitutes a normal comparison. Five groups of rats were used: SHR/NCrl spontaneously hypertensive and WKY/NCrl Wistar/Kyoto rats from Charles River; SD/NTac Sprague Dawley and WH/HanTac Wistar rats from Taconic Europe; and WKY/NHsd Wistar/Kyoto rats from Harlan. DNA was analyzed to determine background differences in the strains by PCR genotyping of eight highly polymorphic microsatellite markers and 2625 single nucleotide polymorphisms (SNPs).

**Results:**

Compared to appropriate comparison strains (WKY/NHsd and SD/NTac rats), SHR/NCrl showed ADHD-C-like behavior: striking overactivity and poor sustained attention. Compared to WKY/NHsd rats, WKY/NCrl rats showed inattention, but no overactivity or impulsiveness. WH/HanTac rats deviated significantly from the other control groups by being more active and less attentive than the WKY/NHsd and SD/NTac rats. We also found substantial genomic differences between the WKY/NCrl and WKY/NHsd rats for eight short tandem repeat loci and 2625 SNPs. About 33.5 percent of the genome differs between the two WKY rat substrains, with large stretches of divergence on each chromosome.

**Discussion:**

These data provide solid behavioral and genetic evidence that the WKY/NCrl and WKY/NHsd rats should be considered as separate substrains. Moreover, the behavioral features of the WKY/NCrl rat indicate that it should be a useful model for ADHD-PI, the primarily inattentive subtype of ADHD. The SD/NTac and the WH/HanTac rats show significant genetic and/or behavioral differences from WKY/NHsd rats and appear not to be appropriate controls in studies using the SHR/NCrl. The present results support the conclusion that SHR/NCrl is the best validated animal model of ADHD-C. The overactivity, impulsiveness and deficient sustained attention of the SHR/NCrl strain are independent behaviors. Thus, overactivity does not account for this strain's impulsiveness and deficient sustained attention. Finally, the present study shows that great care has to be exercised to select the model and comparison groups.

## Background

DSM-IV [1] identifies three subtypes of Attention-Deficit/Hyperactivity Disorder: ADHD predominantly inattentive type (ADHD-PI) if at least six symptoms of inattention, but fewer than six symptoms of hyperactivity-impulsiveness, have persisted for at least 6 months; ADHD predominantly Hyperactive-Impulsive Type (ADHD-HI) if at least six symptoms of hyperactivity-impulsiveness, but fewer than six symptoms of inattention, have persisted for at least 6 months; and ADHD combined type (ADHD-C) if at least six symptoms of inattention and at least six symptoms of hyperactivity-impulsiveness have persisted for at least 6 months. Children with ADHD-PI are often non-hyperactive, rather dreamy, and inert children [2]. Their attention problems are non-specific and related to deficient sensory processes, and poorly focused attention. ADHD-C is more typical amongst boys than girls and ADHD-PI is more typical amongst girls than boys [3]. The inattention of ADHD-C includes difficulty in sustaining attention, distractibility, lack of persistence, and disorganization. Their hyperactivity and impulsiveness includes excessive motor activity and impulsive ('cannot wait') responding. Overall, ADHD affects an estimated 8–12% of children [4]. Moreover, ADHD can persist into adulthood [5], where it affects an estimated 4% of the population [6,7].

Animal models are helpful in medical research because they have simpler nervous systems, more easily interpreted behaviors, more homogeneous genetics, more easily controlled environment, and a greater variety of interventions available [8]. The diagnosis of ADHD is behaviorally based; therefore, validation of animal models must be based in behavior. An ADHD model must mimic the fundamental behavioral characteristics of ADHD (face validity), conform to a theoretical rationale for ADHD (construct validity), and predict aspects of ADHD behavior, genetics, and neurobiology previously uncharted in clinical settings (predictive validity) [8-10].

In many instances, the rat is the most appropriate experimental model of human disease. The large number of inbred rat models and the vast amount of physiological, behavioral, biochemical, cellular, pharmacological, and toxicological data provide a superb platform on which to build the genetic and genomic tools and resources to delineate the connections between genes, biology and disease [11].

The spontaneously hypertensive rat (SHR) is the best validated animal model of ADHD-C. These rats show hyperactivity, impulsiveness and deficits in sustained attention [8,9,12-15]. The control group is usually the Wistar Kyoto Rat (WKY) as this rat is the progenitor strain and its behavior is closely similar to that of other strains when tested in well-controlled operant tasks [8,9].

It is known that the phenotypic expression of the hyperactivity trait is independent of blood pressure status in these SHR and WKY. The high spontaneous activity level of the SHR is not reduced by prevention of the development of hypertension in the young SHR. Nor could high activity levels be induced in the WKY either by acute elevation of the blood pressure by drugs or during the chronic hypertension induced in the WKY and other normotensive strain by means of renal artery constriction [16,17].

We have suggested that ADHD-C and ADHD-PI are two separate disorders probably with separate etiology [18-21]. It is known that commercially available SHR and WKY strains are genetically heterogeneous, probably because they had been separated before they became fully inbred [22-25]. Consequently, rats with different genetic backgrounds were selected for this study. We will show that one of these WKY groups (WKY/NCrl) deviate from normal control rats and may serve as an animal model of ADHD-PI with significant behavioral and genomic differences from the SHR/NCrl and the WKY/NHsd.

## Methods

### Animals

A total number of 44 male rats participated in the behavioral and genetic studies. These included 8 Spontaneously Hypertensive (SHR/NCrl) and 12 Wistar/Kyoto (WKY/NCrl) rats from Charles River (Sulzfeld, Germany); 8 SD/NTac (aka NTac:SD) Sprague Dawley and 8 WH/HanTac (aka HanTac:WH) Wistar Hannover GALAS rats from Taconic Europe, Ry, Denmark; as well as 8 WKY/NHsd Wistar/Kyoto rats from Harlan Europe (Blacktorn, Bicester, UK). Samples of DNA from 2 additional male WKY/NHsd rats from Harlan USA (Indianapolis, IN) were also used as references for the genetic studies. At the start of behavioral testing, the rats were ~4 weeks old and experimentally naïve. Young rats were required, as ADHD primarily is a child and adolescent disorder. The US rats were not tested behaviorally.

At the University of Oslo, the rats were housed individually in 41 × 25 × 25 (length × width × height) cm transparent cages and had free access to food (RM3 (E) from Special Diet Services, Witham, Essex CM8 3AD, UK). The rats had access to water at all times before the habituation session. Starting following completion of the habituation session, the rats were deprived of water for 21 hr a day; this is a moderate, but sufficient deprivation for motivating the animal. The temperature in the housing area was ~24°C. The light in the housing area was on from 0700 to 1900 hours. The behavioral training took place between 1000 and 1330 hours seven days a week.

The study was approved by the Norwegian Animal Research Authority (NARA), and was conducted in accordance with the laws and regulations controlling experiments/procedures in live animals in Norway and the European Union's Directive 86/609/EEC.

### Behavioral apparatus

Sixteen Campden Instruments operant chambers were used in the study. The animal working space in eight of the chambers was 25 × 25 × 30 (height) cm and 25 × 25 × 20 (height) cm in the other eight chambers. A fan producing a low masking noise and the 2.8-W house light were on during the entire experimental session.

During training sessions, either one or both retractable levers were used (below). A 2.8-W cue light was located above each lever. The rats' response consisted of pressing one of the levers with a dead weight of at least 3 g to activate a micro-switch. The reinforcers (0.01 ml tap water) were delivered by a liquid dipper located in a small recessed cubicle with a 2.8-W cue light that lit up when a reinforcer was presented. A 7 × 5 cm transparent plastic lid separated the cubicle from the rat's working space. The rat could easily open the lid with a light push with the nose or paw. Each chamber was ventilated and placed in a sound-resistant outer housing. A computer and an online system (SPIDER, Paul Fray, Ltd., UK) recorded the behavior and scheduled reinforcers (drops of water).

Before the initiation of the study, the rats were assigned a chamber (1 through 16) and time of testing (1000 or 1200 hours) in a randomized and balanced way. The rat was returned to its living cage after each session and immediately given free access to water for 60 min.

### Response acquisition

The training period started with a single 30-min habituation session. During the habituation session, the lid between the working space and the reinforcement cubicle was kept open. The house light was on, but no lever was present, no cue light above any lever was lit, and water was not delivered.

The habituation session was followed by two 30-min dipper training sessions. The lid was kept open, no levers were present, and the house light was on, but the cue lights above the levers were not lit. The computer delivered water on the average every 10 s independent of the rat's behavior (a variable-time schedule). Each water delivery was accompanied by the turning on of the cue light in the small recessed cubicle.

In the next two sessions, the rat was trained to open the lid to gain access to the water. The lid was not taped open, no levers were present, and the lights above the levers were not activated. The house light was on. Each lid opening was followed by a presentation of a single drop of water. The cue light in the recessed cubicle was turned on when water was present.

During the subsequent two sessions, lever responding was shaped by the method of successive approximations [26]. During the first of these sessions, the rats learned to press the left lever in order to receive a reinforcer immediately following every press. The cue light above the left lever was now lit the entire session. The right lever was retracted into the wall and the light above the right lever was off. On the second session, the right lever was activated and the left lever retracted. During this session the light above the right lever was lit the entire session. The house light was on during both sessions. Following this shaping procedure, the animal had acquired the appropriate lever-pressing behavior.

From that point on, both levers were present. The light above the levers shifted randomly. The light stayed lit above a lever for as long as it was the correct lever. This was the discriminative stimulus showing the rat which lever it had to press in order to receive a reinforcer. A concurrent extinction schedule was present on the wrong lever. There was never any light above the extinction lever. Thus, the present task was a simultaneous visual discrimination task. The first four of these sessions lasted for 30 min and the reinforcers were delivered following every correct lever press. Then followed a single session when the reinforcers were delivered according to a 15-s variable-interval schedule where the time between reinforcers ranged from 1 to 120 s in a randomized fashion. Whenever an interval had elapsed, the reinforcer was delivered immediately following the first correct response.

### Final schedule

The simultaneous visual discrimination task was used for testing behavioral characteristics of the various groups (see Additional File 1). An unpredictable 180-s variable-interval schedule was in effect for 90 min on the correct lever (signaled by a constantly lit cue light above this lever) from session 13 (calculated from the initial habituation session) until the study was finished. Inter-reinforcer times ranged from 6 to 719 s in a randomized fashion with a skewed distribution modeled after the "Harvard golden tape" [27]. There was neither any external stimulus signaling that a reinforcer was programmed, nor any external stimulus signaling the time since the last response. A concurrent extinction schedule (never associated with any cue light) was present on the wrong lever. The house light was lit the entire session. Concurrent schedules of reinforcement are schedules of reinforcement that are simultaneously available to an animal subject or human participant, so that the subject or participant can respond on either schedule [26].

### Behavioral measures

Each 90-min session with the final reinforcement schedule was divided into five 18-min segments (parts) in order to monitor intra-session changes in the behavior. For each segment, the total number of presses on the correct and incorrect levers as well as number of reinforcers delivered were recorded. Time between consecutive correct responses (inter-response time, IRT) was also recorded.

The total number of lever presses is an expression of the *general activity *level and therefore a measure of degree of activity. The percent choice of the correct lever when the reinforcers are delivered infrequently is a measure of *sustained attention*. The number of responses with short IRTs (<0.67 s) is used as a measure of degree of *impulsiveness *(cannot hold back a response even when it is an unnecessary one) [9,14,15].

The data were processed by univariate and multivariate analyses of variance (ANOVAs and MANOVAs, respectively) with the Statistica 7.1 program [28]. Within-subject variables are session (every third from 13 through 25) and within-session segment. Group is a between-subject variable. Stable state behavior, means of the final 6 sessions from 22 through 27, was analyzed by ANOVAs followed up by Newman-Keuls test for post hoc evaluations was used for computing approximate probabilities of group differences [28].

### Genetic methods

#### Simple Sequence Length Polymorphism (SSLP) genotyping

In order to determine whether there was any evidence for genetic differences between the WKY/NCrl and WKY/NHsd rats, we genotyped 8 Simple Sequence Length Polymorphisms (SSLPs) that were expected to be highly polymorphic between SD/NTac, WH/HanTac, WKY/NHsd, and SHR/NCrl rats. The SSLPs were amplified using Polymerase Chain Reaction (PCR) on DNA samples from 8 rats in each strain that were purified using the MasterPure kit (Epicentre Biotechnologies, Madison, WI). Primers used were based on the Rat Genome Database records for specific SSLP sequences. The SSLPs chosen interrogated rat chromosomes 1, 2 and 3 at approximately every 60 megabases, and included D1MIT32, D1RAT193, D1RAT196, D2RAT6, D2RAT88, D2RAT171, D3MGH16, and D3MIT13. The PCR products of these reactions were resolved on a 4% agarose/ethidium bromide stained gel by electrophoresis (80 V, 1 h) and compared to the database of expected SSLP sizes for different rat strains available on the Rat Genome Database [25,29].

#### Single Nucleotide Polymorphism (SNP) genotyping

We next sought to estimate the total amount of genomic divergence between the WKY/NCrl and WKY/NHsd rats obtained from the two European sources, and also compare them with WKY/NHsd rat DNA samples from a US supplier (Harlan, Indianapolis, IN) and SHR/NCrl rats. This was accomplished using a whole genome SNP array containing probes for >5,000 SNPs (Targeted Genotyping Rat Panel 1.0 5 K, Affymetrix). DNA samples (2 ug per sample) were obtained from the WKY/NHsd rats (UK, n = 2), the WKY/NHsd rats (US, n = 2), the WKY/NCrl rats (n = 4), and the SHR/NCrl rats (n = 2). These DNAs were processed according to the Affymetrix GeneChip Scanner 3000 Targeted Genotyping System User Guide using the Targeted Genotyping Rat Panel 1.0 5 K Kit. The labeled and tagged DNAs were hybridized to Universal 5 K Tag Arrays at 39°C for 16 h. The arrays were then washed and stained using the TrueTag_Chip_Wash_R7_450 protocol on an Affymetrix Fluidics Station 450. The arrays were scanned using an Affymetrix GeneChip Scanner 3000 and analyzed with Affymetrix GeneChip Targeted Genotyping Analysis Software (TGAS).

## Results

### Behavioral results

#### General

**Figure 1:**
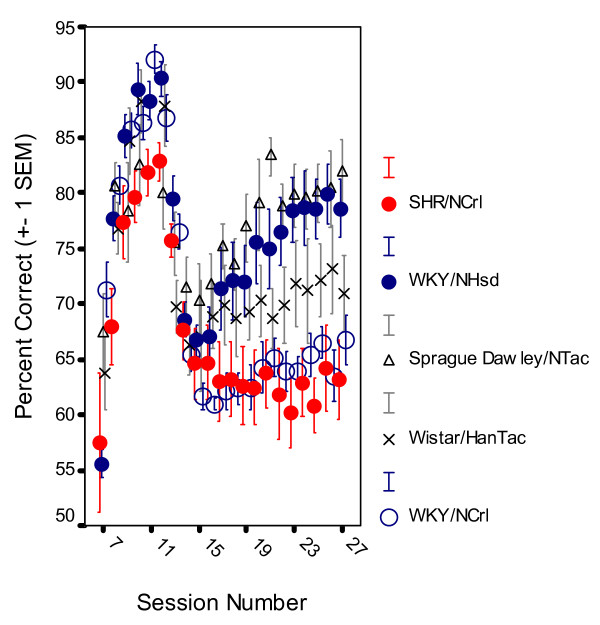
Development of sustained attention, choice of the correct lever in percent of all lever presses, by SHR/NCrl, WKY/NHsd, SD/NTac Sprague Dawley, WH/HanTac Wistar, and WKY/NCrl strains. The final schedule was introduced at session 13 (Means ± 1 SEM).

As is the case in children with ADHD [30,31], the symptoms developed with time, but differently for the different groups and behaviors. All five groups learned which lever was the correct one. All correct responses were reinforced from session 7, when both levers were first available, through session 12. During these sessions, all groups quickly improved their discrimination behavior reaching between 80 and 95% choice of the correct lever within session 12 (Figure 1). It is noteworthy that WKY/NCrl was the group with the non-significant, but still highest percent correct lever choice, slightly more than 90%, at session 12.

The final schedule was installed on session 13. The percentage correct lever choice decreased substantially in all groups during sessions 13 through 15.

**Figure 2:**
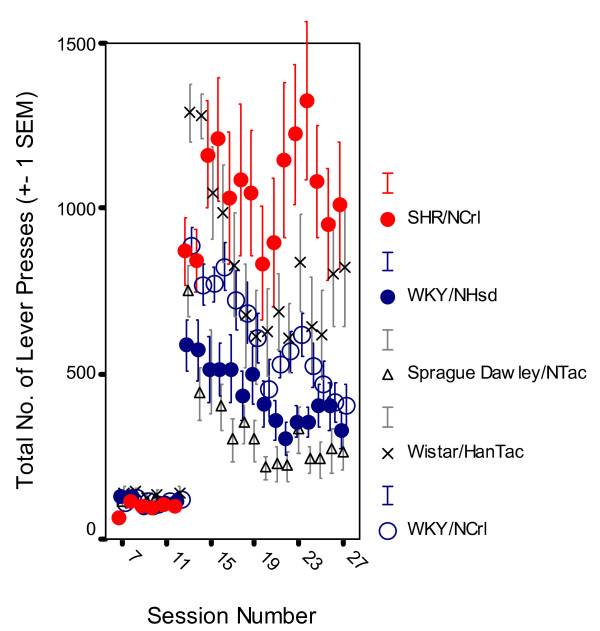
Level of activity, the total number of lever presses, by SHR/NCrl, WKY/NHsd, SD/NTac Sprague Dawley, WH/HanTac Wistar, and WKY/NCrl strains. The final schedule was introduced at session 13 (Means ± 1 SEM).

When behavior had stabilized on the final schedule at session 22, the male SHR/NCrl rats showed poor sustained attention (Figure 1; Additional File 2) and striking overactivity (Figure 2). The WKY/NCrl rats showed as poor sustained attention as the SHR/NCrl rats with just a slight increase in lever-pressing activity (Additional File 3). The WKY/NHsd and the SD/NTac rats showed closely similar, normal behavior. The behavior of the WH/HanTac rats deviated significantly from the other control groups.

#### Sustained attention

Neither SHR/NCrl, WKY/NCrl, nor WH/HanTac rats regained the good performance shown before the introduction of the final schedule at session 13 (Figure 1). The MANOVA of the behavior starting with session 13 through 25 showed main effects of group, *F(4,39) = 6.918, p < 0.001)*; session, *F(4,36) = 19.507, p < 0.001*; within-session segment, *F(4,36) = 2.850, p < 0.04*; group x session interaction, *F(16,111) = 3.623, p < 0.001*; group x within-session segment interaction, *F(16,111) = 2.220, p < 0.01*; but no significant 3-way group x session x segment interaction effect, *F(64,96) = 1.339, p > 0.09*. MANOVA of stable state behavior, means of the final 5 sessions from 22 through 27, showed a main effect of group, *F(4,39) = 9.386, p < 0.001)*. MANOVA of stable state behavior, the means of the final 5 sessions from 22 through 27, showed a main effect of group, *F(4,39) = 9.386*, *p <   0.001*). Follow-up Newman-Keuls tests showed that SHR/NCrl rats were significantly poorer performers than all the other groups except for the WKY/NCrl group (*ps < 0.025*). WKY/NCrl rats were poorer performers than   WKY/NHsd and SD/NTac rats (*ps < 0.005*). There were no significant   differences between WKY/NHsd, SD/NTac rats and WH/HanTac rats (*ps > 0.06*).

#### Overactivity

A pronounced overactivity was seen in SHR/NCrl from session 13 on. Also WH/HanTac rats had a relatively high rate of lever pressing (Figure 2). The activity levels of all groups except for SHR/NCrl, gradually declined by session. The MANOVA of the behavior starting with session 13 through 25 showed main effects of group, *F(4,39) = 9.544, p < 0.001)*; session, *F(4,36) = 13.367, p < 0.001*; within-session segment, *F(4,36) = 40.778, p < 0.001*; group x session interaction, *F(16,111) = 2.824, p < 0.001*; group x within-session segment interaction, *F(16,111) = 2.390, p < 0.005*; and a significant 3-way group x session x segment interaction effect, *F(64,96) = 2.623, p < 0.001*. MANOVAs of stable state behavior showed a main effect of group, *F(4,39) = 9.053, p < 0.001)*. Follow-up Newman-Keuls tests showed that the SHR/NCrl group pressed the levers more than all the other groups (*ps < 0.025*). In addition, WH/HanTac rats were more active than SD/NTac rats (*p < 0.02*). There was no other significant difference between groups.

#### Impulsiveness

**Figure 3:**
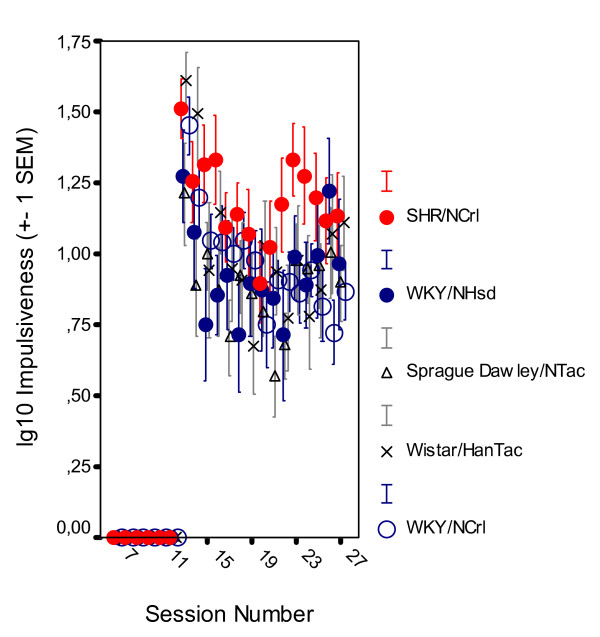
Level of impulsiveness, responding within 0.67 s following the previous lever press, by SHR/NCrl, WKY/NHsd, SD/NTac Sprague Dawley, WH/HanTac Wistar, and WKY/NCrl strains following log10 transformation. The final schedule was introduced at session 13 (Means ± 1 SEM).

The SHR/NCrl group was somewhat more impulsive, responded within 0.67 s since the previous lever press although such a lever press was almost never reinforced, compared to the other groups (Figure 3). The group difference was relatively moderate. The MANOVA for the behavior starting with session 13 through 25 showed no significant main effects of group, *F(4,39) = 0.9, p > 0.40)*; but session, *F(4,36) = 20.446, p < 0.001*; and within-session segment, *F(4,36) = 45.511, 0.001 *effects were significant. There was a group x session interaction, *F(16,111) = 1.849, p < 0.04*; but no other interaction effect. MANOVAs of stable state behavior showed no main effect of group, *F(4,39) = 1.039, p > 0.40)*. This was confirmed by follow-up Newman-Keuls tests showing no significant difference between groups, *ps > 0.30*.

### Genetic differences between WKY/NCrl and WKY/NHsd rats

**Figure 4:**
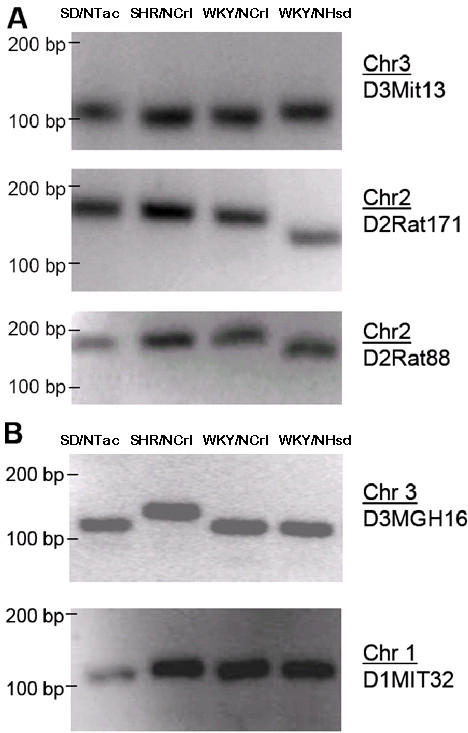
Genotypic differences between SD/NTac Sprague Dawley, SHR/NCrl, WKY/NCrl, and WKY/NHsd strains. (A) For three SSLPs the WKY/NCrl products are the same size as SHR/NCrl. (B) Other SSLPs are the same size for WKY/NCrl and WKY/NHsd (e.g., D3MGH16), indicating that the at least some of the WKY/NHsd background may still be present in the WKY/NCrl.

*SSLP results*. The first step was to examine if there were any genetic differences between the WKY/NCrl and WKY/NHsd rats. Eight SSLPs that are highly polymorphic between all five strains were genotyped. Striking differences between the WKY/NCrl and WKY/NHsd SSLP product sizes were seen (Figure 4). For three SSLPs (Figure 4A), the WKY/NCrl products were the same size as the SHR/NCrl rats (although this in itself could not be taken as evidence of an SHR-WKY intercross, since other strains not tested could also show the same size product). Other SSLPs were the same size in both WKY/NCrl and WKY/NHsd rats (D3MGH16 and D1MIT32, Figure 4B).

### Whole genome SNP genotyping

Given the suggestive results from 8 SSLPs interrogating chromosomes 1, 2 and 3, the second step was to estimate the total amount of genomic divergence or similarity between the rat strains using whole genome SNP arrays. One of the 10 Tag Arrays was observed to have a manufacturing defect, and the data from that sample discarded. For the remaining 9 samples, the overall call rate for a set of quality control SNPs (the QC call rate) was 93.9% and the overall SNP call rate 97.1%. A total of 5296 out of 5455 SNPs on these 9 Tag Arrays passed QC filtering and were examined further. In order to help further reduce the possibility of genotyping error inflating estimates of genomic divergence, we next filtered the SNPs to 2625 high-performing SNPs that generated genotype calls in every sample with an extremely high confidence level.

**Figure 5:**
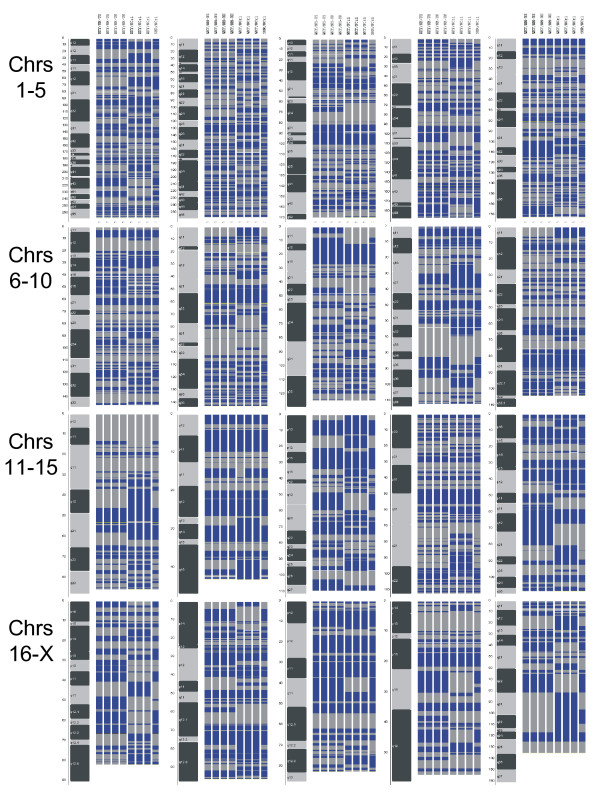
SNP array data confirms genetic divergence of WKY strains. Multiple rat DNA samples from the WKY/NHsd, WKY/NCrl, and SHR/NCrl rats used in the behavioral studies were analyzed using a whole-genome Rat SNP array containing approximately 5,000 SNPs. The data were cleaned and displayed on cytogenetic maps of the rat genome. Blue = BB; Gray = AA; White = AB. Columns 1–2 WKY/NHsd-US; columns 3–4 WKY/NHsd-UK; columns 5–7 WKY/NCrl; column 8 SHR/NCrl. Note that all the WKY/NHsd rats appear isogenic, as do most of the WKY/NCrls, but they do not match each other or the SHR/NCrl completely.

Concordance rates for comparisons between isogenic rats from the same source provide an estimate of genotyping accuracy. The average concordance for all pairwise comparisons of WKY/NHsd rat DNA obtained from either UK or US sources was >99.94% for the set of 2625 high performing SNPs. Among the WKY/NCrl rats, the average pairwise concordance was 99.5%. Thus, both sets of biological replicates showed a high conservation of sequence. In contrast, comparisons between the WKY/NCrl rats and the WKY/NHsd rat (UK or US) indicated a much greater than expected degree of divergence, with average concordance rates of 66.5%. Inspection of the genomic regions harboring SNPs which were discordant between the WKY/NHsd and the WKY/NCrl DNAs revealed large stretches of divergence on every chromosome (Figure 5). These data indicate a large degree of genetic divergence has arisen between WKY/NCrl and WKY/NHsd rats.

Comparisons of SHR/NCrl genotyping data with the WKY/NHsd and WKY/NCrl rat were also informative. Overall, a total of 276/2625 SNPs (10.5%) did not match in comparisons between the SHR/NCrl and WKY/NHsd data. The vast majority of these SNPs (274/276) also did not match in comparisons between WKY/NHsd and WKY/NCrl data. However, of the 276 SNPs that did not match in comparisons of WKY/NHsd and SHR/NCrl rats, almost all (275/276) did match in comparisons of the WKY/NCrl and SHR/NCrl rats. Overall, the WKY/NCrl rats were genetically more similar to the SHR/NCrl rats (76.6% genotypic concordance) than they were to the WKY/NHsd rats (66.5% concordance).

## Discussion

The present study provides behavioral and genotyping data to support the suggestion that the WKY/NCrl rat is a suitable animal model of the inattentive subtype of ADHD (ADHD-PI). This provides researchers with a new tool for studying the neurobiology and genetics of ADHD-PI. Such a model has, heretofore, been unavailable. Our findings are consistent with the fact that the WKY/NCrl and WKY/NHsd substrains had been separated before they became fully inbred [22-24].

The present, as well as other recently published results [9,14,15], show that overactivity, impulsiveness and deficient sustained attention of the SHR/NCrl strain are independent behaviors that may be affected differently by drugs. Further, the present study shows that the WKY/NHsd rat is inattentive without being overactive. Thus, overactivity does not account for impulsiveness and deficient sustained attention in either SHR/NCrl, or in WKY/NHsd rats.

Compared to WKY/NHsd and SD/NTac rat, SHR/NCrl rats showed striking overactivity and poor sustained attention. This result is in accordance with previous studies [8,9,14,15]. In addition, WKY/NCrl rats showed poor sustained attention, but no overactivity or impulsiveness. This result has not been described before.

There were substantial genomic differences between the WKY/NCrl and WKY/NHsd rats for eight SSLP loci and 2625 SNPs. We estimate that 33.5% of the genome differs between the two rat strains, with large stretches of divergence on each chromosome. These data provide solid evidence that the WKY/NCrl and WKY/NHsd rats should be considered as separate strains.

Our genomic results for the SHR/NCrl rats also provide some insights into the possible source of the genetic divergence between the WKY/NHsd and WKY/NCrl rats. 10.5% of the SNPs did not match in comparisons between the SHR/NCrl and WKY/NHsd rat data. Most of these SNPs (99%) also did not match in comparisons between the WKY/NHsd and WKY/NCrl strains. However, of the 276 SNPs that did not match when comparing WKY/NHsd and SHR/NCrl rats, almost all (99%) did match when comparing the WKY/NCrl and SHR/NCrl rats. These data suggest that at least part of the genetic divergence between the WKY/NHsd and WKY/NCrl rats could have been caused by intercrossing with an SHR or SHR-derived rat line. However, the background of the SHR rat alone clearly cannot account for all of the divergence that we have detected.

A more likely explanation of the genetic divergence between the WKY/NHsd and WKY/NCrl rats is that the WKY was not fully inbred when shipped to various breeders [22-24]. The NIH Animal Genetic Resource stock was obtained in 1971 as non-inbred Wistar stock from the Kyoto School of Medicine, Japan. The breeding stock of this strain was distributed before F20, possibly resulting in the emergence of a number of strains or substrains. The WKY/NCrl used in the present study arrived at Charles River in 1974 from NIH at F11. The WKY/NMol, used in several of our previous studies (for a review see [8]), arrived at Møllegaard Breeding Centre, Denmark, from the NIH in 1975 at F13. It is unclear exactly when the Wistar Kyoto rat, later known as WKY/NHsd arrived at Harlan Sprague Dawley, US. The WKY/NCrl appears to now offer the opportunity to evaluate the phenotype and genotype of ADHD-PI in an animal model. It is therefore essential that subline codes are always used in designating this strain [25,29].

The SHR [32] arrived NIH in 1966 at F13 from the Kyoto School of Medicine. It was bred from an outbred Wistar Kyoto male with marked elevation of blood pressure mated to female with slightly elevated blood pressure; brother-sister mating with continued selection for spontaneous hypertension. The SHR/NCrl came to Charles River from NIH in 1973 at F32. There is no evidence for substrain differentiation among SHR stocks from the major commercial suppliers in the USA both respect to phenotype and DNA fingerprints [25,29].

The SD/NTac (NTac:SD), Taconic Sprague Dawley rats, were first obtained in 1970 from the NIH Animal Genetic Resource. The NIH stock originated from Sprague Dawley, Inc. in 1945 and has since been maintained as an outbred closed colony. The WH/HanTac stock was hysterectomy derived at RCC Ltd, Switzerland, in 1989. Genetic drift in RCC's colony of Wistar Hannovers is minimized through the use of the Poiley rotational breeding system and revitalization of the stock with cryopreserved embryos (most recent revitalization completed in 1998). Taconic replaced its former WH stock with the GALAS Wistar Hannover rat in June 2000 [25,29].

The appropriateness of WKY as a control for SHR has systematically been investigated in our laboratory [8]. When using operant schedules of reinforcement, there was no significant behavioral difference between SHR/Mol and SHR/N, but these rats were more active than various comparison groups that did not differ: WKY/Mol, WKY/N, WKHA, WKHT, Wistar/Mol, SD/Mol, hooded PVG/Mol, and offsprings of female DA/OlaHsd crossed with male LEW/NHsd. In some less well-controlled experimental conditions like open fields however, Wistar/Mol and SD/Mol could be as active as SHR/Mol [33]. In conclusion, under well-controlled operant schedules of reinforcement, both the present as well as our previous results [8,9,13,34] indicate that carefully chosen WKY substrains are adequate controls for SHRs.

SD/NTac rats behaved like WKY/NHsd rats in the present study, but the genetic results indicated significant differences between this strain and the WKY/NHsd strain. Thus, Sprague Dawley rats may be a poor control for the SHR in neurobiological studies. Given that the present results indicate that the WH/HanTac rats and WKY/NCrl deviated both genetically as well as behaviorally from the WKY/NHsd, we conclude that the use of these strains as controls for SHRs may also produce spurious neurobiological differences. Thus, WKY/NHsd is the most appropriate control for SHR/NCrl.

There are significant neurobiological differences between the SHR/NCrl and WKY/NHsd rats [35]. Current theories of ADHD relate symptom development to factors that alter learning [21,36,37]. N-methyl-D-aspartate receptor (NMDAR) dependent long term changes in synaptic efficacy in the mammalian CNS are thought to represent underlying cellular mechanisms for some forms of learning [38,39]. Physiological and anatomical aspects of hippocampal CA3-to-CA1 synapses in age-matched SHR/NCrl and WKY/NHsd rats showed functional impairments in glutamatergic synaptic transmission that may be one of the underlying mechanisms leading to the abnormal behavior in SHR/NCrl and possibly in ADHD [35].

## Limitations

As an unavoidable consequence of the need to have rats with different genetic backgrounds, rats had to be purchased from different breeders. Consequently, differences in early-life environments may have contributed to the present strain differences. We have, however, replicated the behavioral differences between SHR/NCrl, WKY/NCrl and WKY/NHsd groups in rats that were bred in our own facility. Thus, early-life environmental differences are unlikely to have played a major role in the presently-observed strain differences.

## Conclusion

The present results suggest that WKY/NCrl is a promising model of ADHD-PI and confirms that SHR may be used as an animal model of ADHD-C. Finally, the present study shows that great care has to be exercised when selecting the model and comparison groups.

## Competing interests

The authors declare that they have no competing interests.

## Authors' contributions

TS found the animal models, designed and carried out the behavioral testing. TD, YZ, FAM and SVF carried out the molecular genetic studies. All authors participated in writing and approved the final manuscript.

## Supplementary Material

Additional file 1 The video shows a normal male WKY/NHsd control rat performing the visual discrimination task. Click here for file

Additional file 2 The video shows a male SHR/NCrl ("ADHD-C") rat performing the visual discrimination task. Click here for file

Additional file 3 The video shows a male WKY/NCrl ("ADHD-PI") rat performing the visual discrimination task. Click here for file
